# Physiology, Metabolomics, and Transcriptomics Reveal Effects of AMF and *Chaetomium globosum* Co-Inoculation on Growth and Medicinal Compounds in *Astragalus membranaceus*

**DOI:** 10.3390/metabo16050313

**Published:** 2026-05-03

**Authors:** Yuxin Gong, Shengnan Liu, Xiaoju Zhao, Weisan Zhang, Jiaotong Li, Mengqi Liu, Xueqi Zhang, Hanqi Jia, Zhonghua Tang

**Affiliations:** 1 College of Chemistry, Chemical Engineering and Resource Utilization, Northeast Forestry University, Harbin 150040, China; 2024120797@nefu.edu.cn (Y.G.);; 2 Key Laboratory of Forest Plant Ecology, Ministry of Education, Northeast Forestry University, Harbin 150040, China; 3 China Resources Sanjiu Traditional Chinese Medicine Inheritance and Innovation Co., Ltd., Jiamusi 154005, China; 4 Bioengineering Institute, Daqing Normal University, Daqing 163712, China

**Keywords:** arbuscular mycorrhizal fungi (AMF), *Chaetomium globosum*, *Astragalus membranaceus*, plant growth promotion, secondary metabolism, flavonoid biosynthesis pathway

## Abstract

**Background/Objectives:** Microbial inoculants effectively alleviate inhibitory factors during plant cultivation; however, the effects and underlying mechanisms of arbuscular mycorrhizal fungi (AMF) and *Chaetomium globosum* on the growth, metabolism, and bioactive compound production of *A. membranaceus* are still poorly understood. **Methods**: In this experiment, different concentrations of *C. globosum* (10^4^, 10^6^, 10^8^ spores/mL) (Q1, Q2, Q3), AMF, and their combined treatments (AQ1, AQ2, AQ3) were applied to *A. membranaceus* seedlings via root irrigation, with an equal amount of sterile water as a control (CK). **Results**: The results showed that: (1) Under single inoculation with *C. globosum*, root colonization rate increased with higher inoculation concentrations, reaching its peak at Q3. Additionally, AQ3 significantly enhanced AMF colonization in *A. membranaceus*, and the presence of *C. globosum* promoted AMF root colonization and expansion. (2) AQ3 significantly enhanced the growth and photosynthesis of *A. membranaceus*, while also demonstrating excellent efficacy in alleviating lipid peroxidation-induced damage. (3) AQ3 treatment led to increased accumulation of major bioactive compounds in *A. membranaceus*, including calycosin-7-glucoside, cycloastragenol, and astragalosides I–III. (4) AQ3 treatment significantly upregulated multiple key structural genes involved in phenylalanine metabolism and flavonoid biosynthesis pathways, including *PAL*, *4CL*, *FLS*, and *CYP75B1*. **Conclusions**: This upregulation enhanced the metabolic flux allocation from L-phenylalanine toward downstream flavonoid metabolites, thereby promoting the accumulation of major flavonoid constituents of *A. membranaceus*, such as galangin and luteolin, in both roots and leaves.

## 1. Introduction

*Astragalus membranaceus* (Fisch.) Bge., a perennial herb in the Fabaceae family, is recorded as a highly valued medicinal plant in the Shennong Ben Cao Jing and remains one of the most widely used herbs in traditional Chinese medicine. *A. membranaceus* contains a variety of bioactive constituents, including polysaccharides, flavonoids, and saponins (notably astragalosides). Traditionally, it has been used for Qi tonification, diuretic purposes, and the promotion of tissue regeneration [1]. Contemporary pharmacological studies further demonstrate that *A. membranaceus* exhibits notable activities in immune regulation, antioxidation, anti-inflammation, and anticancer responses [2]. These properties have contributed to its growing applications in pharmaceuticals, health supplements, and functional food products [3].

*Astragalus membranaceus* is a widely used traditional medicinal plant with high demand due to its rich content of various bioactive compounds, which confer significant therapeutic value. With the growing market demand, the production of *A. membranaceus* has gradually shifted from traditional wild or semi-wild collection to large-scale artificial cultivation. However, excessive use of chemical fertilizers and pesticides during long-term intensive cultivation has contributed to soil degradation and microbial community imbalance, resulting in suppressed plant growth, reduced yield, and decreased accumulation of bioactive compounds, which severely limits the sustainable development of the Astragalus industry [4]. Therefore, there is an urgent need to explore green and efficient cultivation strategies to improve both the yield and quality.

Arbuscular mycorrhizal fungi (AMF) are a group of widely distributed symbiotic fungi that form mutually beneficial relationships with the roots of most terrestrial plants [5]. AMF expand the root absorption area through their hyphal network, thereby enhancing plant uptake of essential mineral nutrients such as phosphorus and nitrogen, while also enhancing tolerance to abiotic stresses, including drought, salt, and heavy metal toxicity [6]. Previous studies have shown that AMF can promote plant biomass accumulation and enhance photosynthetic efficiency [7]. For example, inoculation with AMF significantly promoted the growth and development of *Salvia miltiorrhiza* seedlings [8]. AMF can also regulate the plant’s antioxidant enzyme system and secondary metabolic pathways, improving crop quality and promoting the biosynthesis of functional metabolites [6,9]. Studies have found that AMF treatment increased the content of secondary metabolites such as polyphenols, flavonoids, and anthocyanins in yam (*Dioscorea* spp.) tubers [10]. In tobacco (*Nicotiana tabacum*) and rose (*Catharanthus roseus*), AMF inoculation promoted the synthesis and accumulation of alkaloids [11]. However, the interaction effects between different plant–AMF combinations vary significantly [12], and the mechanisms underlying their impact on the growth and accumulation of bioactive compounds in medicinal plants, especially *A. membranaceus*, remain to be further elucidated.

*Chaetomium globosum* is a common soil fungus with the ability to establish endophytic colonization in plants [13,14]. It can suppress pathogen invasion and improve the rhizosphere microenvironment through the secretion of various extracellular enzymes, plant hormone analogs, and antagonistic metabolites [15]. In addition, the production of ergot-like compounds has been reported to enhance the plant antioxidant defense system, induce metabolic reprogramming, and ultimately promote plant growth [16]. For example, in maize, colonization by *C. globosum* has been shown to influence osmolyte levels, antioxidant enzyme activity, and lipid peroxidation, which in turn improves seedling tolerance to copper stress [17]. Thus, *C. globosum* is considered a promising microorganism for plant growth promotion and biological control [18]. However, its functional role in medicinal plants remains insufficiently explored. In particular, the potential interactions between *C. globosum* and AMF, as well as their combined effects on plant growth and bioactive compound accumulation, are still not well understood. This lack of insight hinders the widespread application of microbial inoculants in the sustainable cultivation of medicinal plants.

Microbial inoculants, as an essential component of modern sustainable agriculture [19], have gradually become a key technology for replacing chemical fertilizers and pesticides due to their environmental friendliness, sustainability, and multi-target regulatory advantages [20]. Combined microbial inoculants often exhibit superior effects in promoting plant growth [21], enhancing stress resistance, and improving quality through the synergistic actions of different functional microorganisms compared to single inoculants [22,23]. However, the interactions between different microorganisms and their comprehensive regulatory mechanisms on the host plant’s metabolic network remain to be further elucidated [24].

Based on this, this study took *A. membranaceus* as the research object and systematically explored the effects of AMF and different concentrations of *C. globosum* on the growth, photosynthesis, antioxidant system, accumulation of medicinal active metabolites and secondary metabolites of *A. membranaceus* and their mechanisms under the conditions of single and combined application.

By integrating physiological measurements with LC–MS-based metabolomics and transcriptomic analyses, this study aimed to elucidate the regulatory mechanisms underlying metabolic reprogramming induced by compound microbial inoculation in *A. membranaceus*. The main novelties of this study are summarized as follows: (1) this work provides the first systematic comparison of single and combined applications of AMF and *C. globosum* at different concentrations on growth performance and medicinal compound accumulation in *A. membranaceus*; (2) it reveals the regulatory mechanisms of compound microbial inoculation on phenolic metabolism in *A. membranaceus* through combined secondary metabolite profiling and transcriptomic analysis; (3) it provides a theoretical basis and practical implications for the application of microbial inoculants in the green and efficient cultivation of medicinal plants.

## 2. Materials and Methods

### 2.1. Inoculum Preparation of AMF and C. globosum

The AMF utilized in this investigation were sourced from the Forest Pathology Laboratory at Northeast Forestry University in China, where the species had been taxonomically classified as *Gigaspora margarita*. The propagation of AMF spores was conducted in cultivation containers measuring 165 mm × 130 mm, with maize (Zhengdan 958 variety) serving as the host crop for fungal multiplication. Prior to planting, maize seeds underwent surface sterilization by immersion in a 5% sodium hypochlorite solution for 8 min, followed by thorough rinsing with sterilized distilled water. These treated seeds were then planted in plastic containers containing autoclave-sterilized soil (sterilization performed at 121 °C for 2 h). The prepared AMF inoculum was introduced into these pots containing the sterilized growth medium and seeds. Cultivation occurred under greenhouse conditions with regular irrigation using tap water. Upon reaching 90 days of growth, watering was discontinued, and plant root systems were harvested for assessment of fungal colonization using the established protocol developed by Koske and Gemma [25]. Subsequently, soil samples were subjected to air-drying, followed by quantification of AMF spores through sucrose density gradient centrifugation as outlined in reference [26]. The experimental protocol stipulated that soil samples must contain a minimum of 25 fungal spores per gram to be considered suitable for the study.

In October 2024, researchers successfully isolated *C. globosum* from the root systems of *A. membranaceus* plants cultivated in the greenhouse facilities of Northeast Forestry University, located in Harbin, Heilongjiang Province, China. For spore propagation, the fungal culture was maintained on potato dextrose agar medium (PDA), with each liter of medium consisting of 200 g of potato extract, 20 g of glucose, and 20 g of agar. The cultivation process occurred at a constant temperature of 25 °C under complete darkness. To harvest the spores, sterile distilled water was used to rinse the culture plates, followed by quantification of spore density using a hemocytometer, ultimately achieving a standardized concentration of 1 × 10^8^ spores per milliliter [14].

### 2.2. Plant Materials and Cultivation Conditions

The experiment used one-year-old *A. membranaceus* seeds, which were planted in seedling pots. When the seedlings reached the four-leaf stage with a central leaf, uniform and healthy seedlings were selected and transplanted into pots (250 mm × 200 mm) filled with a 3:1 soil-sand mixture, with 4 seedlings per pot. The experimental treatments included single and dual inoculations, there were 8 treatment groups: CK (inoculation with sterilized inoculants containing no viable AMF or *C. globosum*), Q1 (single inoculation with *C. globosum* at 10^4^ spores/mL), Q2 (single inoculation with *C. globosum* at 10^6^ spores/mL), Q3 (single inoculation with *C. globosum* at 10^8^ spores/mL), AMF (single inoculation with AMF), AQ1 (simultaneous inoculation with AMF and *C. globosum* at 10^4^ spores/mL), AQ2 (simultaneous inoculation with AMF and *C. globosum* at 10^6^ spores/mL), and AQ3 (simultaneous inoculation with AMF and *C. globosum* at 10^8^ spores/mL).

Growth conditions: The plants were placed in a greenhouse with temperatures ranging from 20 to 26 °C, relative humidity between 65% and 90%, and natural light (Supplementary File S1). Watering was done every 2 days.

The seedlings were placed in pots with a round plastic sheet of the same size as the pot bottom. About half of the sterilized substrate (3:1 soil: vermiculite) was added, and 4 healthy seedlings were transplanted into each pot. AMF inoculum was applied at a rate of 150 spores per pot. Root inoculation was performed by applying 20 mL of *C. globosum* inoculant per pot. The seedlings were harvested 90 days after inoculation with the different treatments.

### 2.3. Assessments of AMF and C. globosum Colonization Rate

The root specimens were thoroughly rinsed to eliminate any adhering soil particles before undergoing a cleaning process in a 10% potassium hydroxide solution (weight/volume) maintained at 90 °C for one hour. Subsequently, the samples were acid-treated with 1% hydrochloric acid (volume/volume) and then subjected to staining with 0.05% aniline blue solution at the same temperature for half an hour. For assessment of colonization patterns, thirty root sections measuring 1 cm each were microscopically examined under 40-fold magnification. The extent of arbuscular mycorrhizal fungi and *C. globosum* colonization within root tissues was quantified through the grid-line intersect technique, with analysis performed across over 100 intersection points [27].

### 2.4. Measurement of Plant Growth and Morphology

Following a 90-day experimental period, six seedlings from each test condition were chosen at random for comprehensive analysis of growth characteristics. Measurements included vertical plant stature (determined from the base to the terminal bud), underground root system extension, biomass in fresh state, and dehydrated tissue mass.

Efficacy (%) = [(Value (treatment) − Value (control))/Value (control)] × 100%

### 2.5. Measurement of Photosynthetic Parameters and Chlorophyll Fluorescence Parameters

Photosynthetic parameters of *A. membranaceus* leaves were measured and calculated using a Li-6400 portable photosynthesis system (LI-COR, Lincoln, NE, USA) between 9 and 11 AM on sunny days. Chlorophyll fluorescence parameters of *A. membranaceus* leaves were measured in the evening using a portable PAM-2500 chlorophyll fluorescence system (Zeqi Technology, Shanghai, China). Six pots from each treatment group were randomly selected for the measurements.

### 2.6. Analysis of Malondialdehyde, Superoxide Anion, and Antioxidant Enzyme Activities

Quantitative assessments were conducted for malondialdehyde (MDA) content, superoxide radical (O_2_^.–^) levels, along with the enzymatic activities of catalase (CAT), peroxidase (POD), superoxide dismutase (SOD), and ascorbic acid peroxidase (APX). A fresh plant specimen weighing exactly 0.2 g was carefully measured. To this sample, 5 milliliters of phosphate buffer solution (pre-chilled to maintain a pH of 7.8) was added. The mixture was thoroughly homogenized using an ice-cold mortar and pestle, then subjected to centrifugation at 4 °C with a rotational speed of 8000 revolutions per minute for half an hour. This procedure yielded the crude enzyme extract. CAT enzymatic activity was quantified following established protocols from reference [28], with calculations derived from absorbance measurements taken at 240 nm wavelength. For POD activity evaluation, the guaiacol oxidation technique was implemented, monitoring absorbance changes at 470 nm in 30 s intervals for three minutes following enzyme introduction. SOD activity assessment utilized the photochemical reduction method involving nitrogen blue tetrazolium (NBT). The measurement of APX activity involved tracking absorbance variations at 290 nm over a three-minute observation window. The concentrations of MDA and O_2_^.–^ were measured following the experimental procedures outlined in reference [29]. Three biological replicates were examined for every experimental condition, with each replicate consisting of a composite sample obtained from three randomly chosen seedlings.

### 2.7. Sample Preparation and LC-MS Analysis

A quantity of 0.2 g of dehydrated specimens underwent dissolution in 10 milliliters of methanol. The mixture was subjected to homogenization at 70 Hz for a duration between 6 to 8 min, followed by ultrasonic treatment (250 W, 40 kHz) for 60 min. After centrifugation at 8000 rpm for 10 min, the clear liquid portion was separated. The remaining solid material underwent secondary extraction using 8 milliliters of methanol, with the resulting supernatant being merged with the initial extract. The combined solution was then evaporated under vacuum conditions, redissolved in 1 milliliter of 70% methanol solution, and passed through a 0.45 μm nylon membrane. Metabolic profiling was conducted employing a Waters Xevo G2 QTOF mass analyzer integrated with an ultra-performance liquid chromatography system, operating in positive ionization mode (mass range 50–1000 Da) following standard procedures. Identification of chemical constituents was achieved by comparing retention characteristics, molecular ions, significant secondary ions, and fragmentation behaviors with reference standards. Quantitative assessment of individual compounds was performed through peak area integration using Thermo Scientific Xcalibur software (version 4.2). The experimental procedure was repeated across three independent biological samples [30].

### 2.8. Transcriptome Sequencing and Analysis

Samples of *A. membranaceus* subjected to various experimental conditions exhibited notable morphological and functional variations. These specimens underwent thorough rinsing with ultrapure water, blot drying using filter paper, rapid freezing in liquid nitrogen for half an hour, and preservation at −80 °C [31]. Total RNA extraction, cDNA library construction, and high-throughput sequencing were performed by Metware Biotechnology Co., Ltd. (Wuhan, China) following standard protocols. Briefly, total RNA was extracted from the samples, and its concentration and integrity were assessed prior to library construction. Subsequently, mRNA was enriched, fragmented, and reverse-transcribed into cDNA, followed by end repair, adapter ligation, and PCR amplification to complete the sequencing library construction.

After sequencing, raw sequencing data were filtered using fastp to generate clean reads. The clean reads were then aligned to the reference genome using HISAT2 [32]. Transcript assembly was performed in a reference guided manner using StringTie, and novel transcripts were identified by comparing the assembled transcripts with the reference annotation. The reference genome assembly used in this study is available in the Integrated Medicinal Plantomics (IMP) database under the version name Ame1. Gene level read counts were obtained using featureCounts, and gene expression abundance was normalized as fragments per kilobase of transcript per million mapped reads (FPKM). Differential expression analysis between groups was conducted using DESeq2 based on raw count data to identify differentially expressed genes (DEGs) [33,34]. *p* values were adjusted using the Benjamini–Hochberg method to control the false discovery rate (FDR).

### 2.9. Statistical Analysis

The data analysis was carried out with SPSS version 27.0.1.0. Prior to implementing one-way ANOVA and Duncan’s post hoc comparisons, the data’s compliance with parametric test requirements was verified. The Shapiro–Wilk procedure was employed to examine normal distribution, with all variables demonstrating *p*-values exceeding 0.05, confirming normal distribution. Variance homogeneity was evaluated through the Brown–Forsythe method, with all *p*-values surpassing 0.05, meeting the variance equality criterion. Where assumptions were violated, the nonparametric Kruskal–Wallis test was substituted. Graphical representation of ANOVA outcomes was created using Origin software (version 10.1.0.178). Principal component analysis was conducted via the web-based platform MetaboAnalyst 6.0. Heatmap visualization of differentially expressed metabolites was generated using R software (version 4.4.2). Metabolic pathway enrichment analysis of differential metabolites (DEMs) was performed using the Kyoto Encyclopedia of Genes and Genomes (KEGG). The Metware Cloud Tools (https://cloud.metware.cn) and Adobe Illustrator CC 2019 are used for bioinformatics analysis and visualization.

## 3. Results

### 3.1. Colonization of AMF and C. globosum in A. membranaceus Roots

**Figure 1:**
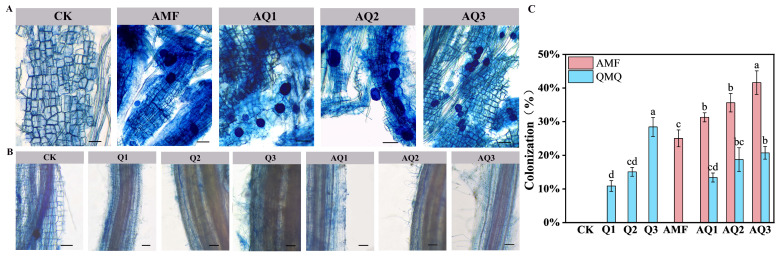
Colonization of AMF and *C. globosum* in *A. membranaceus* roots. (**A**) Colonization of AMF in different treatment groups, from left to right: CK, AMF, AQ1, AQ2, AQ3; (**B**) Colonization of *C. globosum* in different treatment groups, from left to right: CK, Q1, Q2, Q3, AQ1, AQ2, AQ3; (**C**) Colonization rate of AMF and *C. globosum.* Groups marked with different lowercase letters (a, b, c, …) are significantly different, while groups sharing the same letter are not significantly different. Scale bars: 100 µm.

Microscopic observations showed that both single and dual inoculation with AMF and *C. globosum* successfully colonized the roots of *A. membranaceus*, although colonization rates differed among treatments. Under single inoculation with different concentrations of *C. globosum*, root colonization peaked in the Q3 treatment, reaching 28.43%. In dual inoculation treatments, *C. globosum* colonization rates in AQ1 and AQ2 were 13.40% and 18.73%, respectively, both exceeding those observed under the corresponding single inoculations (10.87% and 15.07%). In contrast, *C. globosum* colonization under AQ3 reached 20.73%, which was lower than that of the corresponding single inoculation (28.43%) (Figure 1B,C). Measurement of AMF colonization revealed that dual inoculation resulted in significantly higher colonization rates than single AMF inoculation. AMF colonization was highest under the AQ3 treatment, reaching 41.60%, representing a 66.17% increase compared with the control (CK) (Figure 1A,C). The complete ANOVA results, including the exact *p*-values, Cv, df, and F values, are provided in the attached Supplementary File S2.

### 3.2. Effects of Different Microbial Inoculations on Morphological Growth and Medicinal Metabolite Accumulation of A. membranaceus

**Figure 2:**
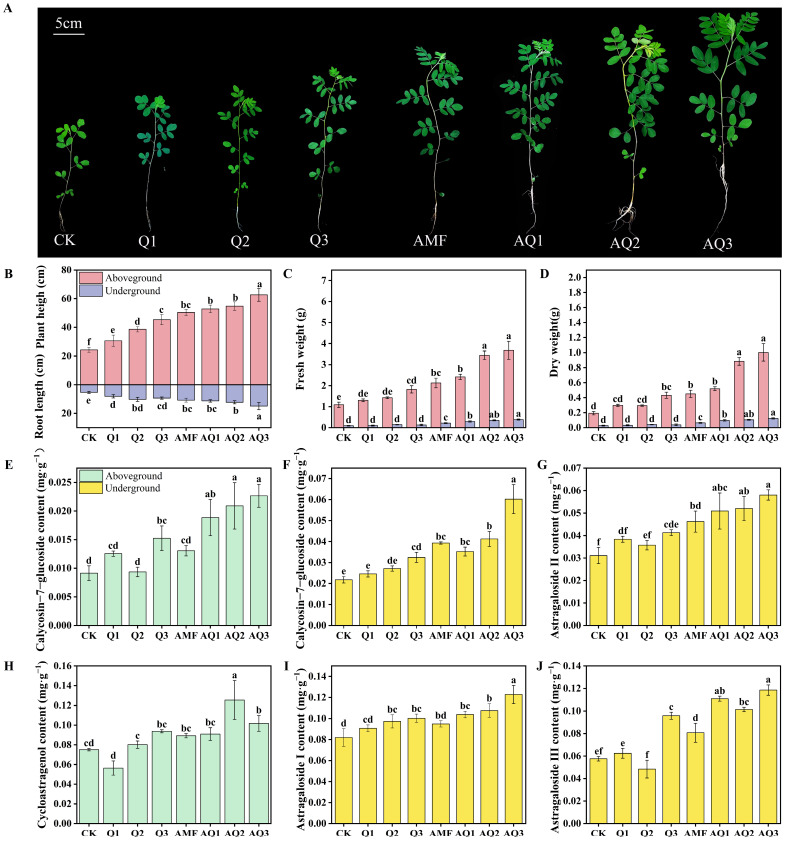
The changes in the growth morphology and the content of medicinal active metabolites of *A. membranaceus* after applying different microbial agents. (**A**) Morphology of *A. membranaceus* plants after application of microbial agent; (**B**) Plant height and root length; (**C**) Fresh weight; (**D**) Dry weight; (**E**) Content of calycosin-7-glucoside in the aboveground part; (**F**) Content of calycosin-7-glucoside in the underground part; (**G**) Content of astragaloside II in the underground part; (**H**) Content of cycloastragenol in the aboveground part; (**I**) Content of astragaloside I in the underground part; (**J**) Content of astragaloside III in the underground part. Groups marked with different lowercase letters (a, b, c, …) are significantly different, while groups sharing the same letter are not significantly different.

The results showed that both single inoculation with AMF or *C. globosum* and their dual inoculation promoted the growth of *A. membranaceus* seedlings to varying extents, resulting in increased plant height, root length, fresh weight, and dry weight compared with the control (CK) (Figure 2A–D). Notably, all growth parameters under dual inoculation treatments were significantly higher than those observed under single inoculation. Among them, the AQ3 treatment exhibited the strongest growth-promoting effect, with plant height and root length increasing by 158.77% and 178.97%, respectively. In the AQ2 and AQ1 treatments, plant height and root length increased by 126.22% and 128.97%, and by 118.10% and 111.21%, respectively (Figure 2B). Among single inoculation treatments, Q3 and AMF showed more pronounced growth-promoting effects. Under Q3, plant height and root length increased by 87.16% and 75.08%, respectively, whereas AMF inoculation resulted in increases of 108.09% and 101.56% (Figure 2B). No significant differences in aboveground biomass were observed under Q1 and Q2 treatments, and root biomass did not differ significantly under Q1, Q2, or Q3 treatments. In contrast, AQ3 markedly increased biomass accumulation, leading to increases of 237.61% (fresh weight) and 419.35% (dry weight) in aboveground biomass and 316.22% (fresh weight) and 347.69% (dry weight) in belowground biomass compared with CK (Figure 2C,D).

LC–MS analysis was conducted to evaluate the effects of microbial inoculation on medicinally active metabolites in *A. membranaceus*. Calycosin-7-glucoside and cycloastragenol were the predominant compounds detected in the aboveground part. Compared with CK, calycosin-7-glucoside content increased by 66.77%, 106.14%, 128.55%, and 147.75% under the Q3, AQ1, AQ2, and AQ3 treatments, respectively (Figure 2E). In contrast, cycloastragenol content increased significantly only under AQ2 and AQ3, reaching a maximum increase of 67.09% under AQ2, while no significant changes were observed under other treatments and a reduction was detected under Q1 (Figure 2H). In roots, calycosin-7-glucoside, astragaloside I, astragaloside II, and astragaloside III were the major active components. No significant differences in calycosin-7-glucoside, astragaloside II, or astragaloside III were observed under Q1 or Q2 treatments, and astragaloside I content was not significantly affected by Q1 or AMF. All four metabolites reached their highest levels under AQ3, with increases of 176.55%, 49.84%, 86.64%, and 105.69%, respectively, compared with CK (Figure 2F,G,I,J).

### 3.3. Effects of Different Microbial Inoculants on Photosynthesis and Chlorophyll Fluorescence in A. membranaceus

The results indicated that both single and dual inoculation with AMF and *C. globosum* significantly enhanced the net photosynthetic rate (Pn), stomatal conductance (Gs), and transpiration rate (Tr) of *A. membranaceus* compared with the control (CK). All three parameters reached their highest values under the AQ3 treatment, with Pn, Gs, and Tr reaching 9.103 μmol·m^−2^·s ^−1^, 0.095 μmol·m^−2^·s^−1^, and 1.890 μmol·m^−2^·s^−1^, respectively. These results demonstrate that AMF and *C. globosum* inoculation promoted photosynthetic activity and transpiration, with the most pronounced effects observed under AQ3. Intercellular CO_2_ concentration (Ci) did not differ significantly from CK only under the Q1 treatment, whereas all other inoculation treatments resulted in significantly higher Ci values. The maximum Ci was observed under AQ2, reaching 283.333 μmol·m^−2^·s^−1^, which represented a 32.60% increase compared with CK (Table S1). The photochemical quenching coefficient (qP) increased significantly only under the AQ3 treatment, showing a 13.64% increase relative to CK, while no significant differences were detected under the remaining treatments. In contrast, non-photochemical quenching (NPQ) increased significantly under AQ2 and AQ3, with increases of 102.91% and 207.56%, respectively. Both the actual photochemical efficiency of PSII (Y (II)) and the electron transport rate (ETR) were significantly enhanced by microbial inoculation compared with CK, and both parameters reached their highest values under AQ3. Specifically, Y (II) and ETR increased by 19.09% and 18.88%, respectively, under AQ3. Moreover, the potential photochemical activity of PSII (Fv/Fo) was significantly enhanced under Q3 and all dual inoculation treatments, increasing by 24.76%, 22.01%, 26.21%, and 24.82% under Q3, AQ1, AQ2, and AQ3, respectively (Table S2).

### 3.4. Effects of Different Microbial Inoculants on Antioxidant Activity and Reactive Oxygen Species Content in A. membranaceus

**Figure 3:**
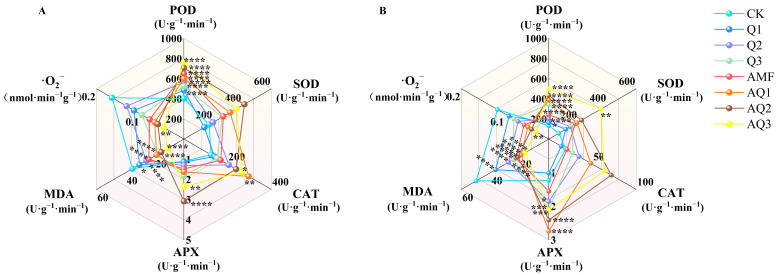
The antioxidant enzyme activity and MDA content of *A. membranaceus* after the application of different microbial agents. (**A**) Aboveground part of plant; (**B**) Underground part of plant. APX: ascorbate peroxidase; CAT: catalase; MDA: malondialdehyde; O_2_^.–^: superoxide anion; POD: peroxidase; SOD: superoxide dismutase. *: *p* < 0.05; **: *p* < 0.01; ***: *p* < 0.001; ****: *p* < 0.0001.

Antioxidant enzymes in plant cells, including superoxide dismutase (SOD), peroxidase (POD), catalase (CAT), and ascorbate peroxidase (APX), convert reactive oxygen species (ROS) into less harmful compounds, thereby contributing to plant adaptation to environmental changes. The activities of these antioxidant enzymes in *A. membranaceus* seedlings were enhanced to varying degrees following inoculation with different microbial agents. Notably, APX, SOD, POD, and CAT activities under dual inoculation treatments were significantly higher than those in the control (CK). POD activity reached its maximum level under the AQ3 treatment, increasing by 89.94% (aboveground) and 275.56% (underground) compared with CK. SOD activity in the aboveground part was highest under AQ2, showing a 140% increase relative to CK, whereas root SOD activity peaked under AQ3 and was approximately 4.16-fold higher than that of CK. The CAT enzyme activity in the aboveground part was highest under AQ1, increasing by 138.48% compared with CK, while no significant enhancement was observed under Q1 or Q3. In roots, CAT activity reached its maximum under AQ2, increasing by 269.48%, whereas Q1 resulted in a significant inhibition, with a 26.21% decrease relative to CK. The APX activity in the aboveground part was significantly enhanced under AQ2 and AQ3, increasing by 180.08% and 114.28%, respectively. In roots, APX activity reached its highest level under AQ1, showing a 122.73% increase compared with CK, whereas Q1 caused a significant suppression, with a 17.69% reduction. Following inoculation with AMF and *C. globosum*, malondialdehyde (MDA) contents were significantly reduced. Under AQ3, MDA content decreased by 68.49% (aboveground) and 67.45% (underground). The superoxide anion (O_2_^.–^) production rate was further evaluated and showed significant reductions in the aboveground part under all inoculation treatments. The lowest O_2_^.–^ production rate in the aboveground part was observed under AQ3 (0.045 ± 0.07 nmol·min^−1^·g ^−1^), representing a 72.07% decrease compared with CK. In roots, no significant change was detected under Q1, whereas AQ3 resulted in the lowest O_2_^.–^ production rate, with a 75.46% reduction relative to CK (Figure 3).

### 3.5. Effects of Microbial Treatments on Phenolic Metabolites in A. membranaceus

**Figure 4:**
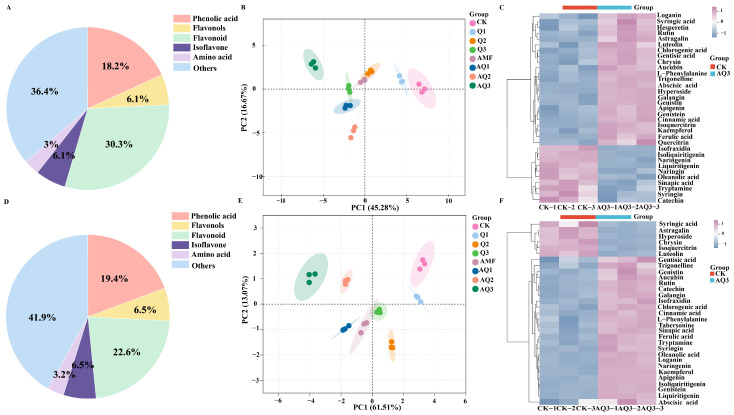
Secondary metabolites of *A. membranaceus* under different microbial agents. (**A**) Metabolites in the aboveground part of plant; (**B**) PCA score plot of the aboveground part of plant; (**C**) Clustering heatmap of the different metabolites of the aboveground part of plant; (**D**) Metabolites in the underground part of plant; (**E**) PCA score plot of the underground part of plant; (**F**) Clustering heatmap of the different metabolites of the underground part of plant.

Using targeted metabolomics analysis, we further investigated the accumulation of secondary metabolites in *A. membranaceus* following microbial treatments. A total of 33 phenolic compounds were identified in leaves by LC–MS, including 10 flavonoids, 6 phenolic acids, 2 isoflavones, 2 flavonols, 1 amino acid, and 12 other compounds. In roots, 31 phenolic compounds were identified, comprising 7 flavonoids, 6 phenolic acids, 2 isoflavones, 2 flavonols, 1 amino acid, and 13 other compounds (Figure 4A,D) (Supplementary File S4).

Principal component analysis (PCA) revealed clear separation among different treatments. In leaves, the first two principal components, PC1 and PC2, explained 45.28% and 16.67% of the total variance, respectively. In roots, PC1 and PC2 accounted for 61.51% and 13.07% of the total variance, respectively, indicating distinct metabolic profiles among treatments (Figure 4B,E).

The control (CK) and AQ3 treatment groups were selected for further analysis. Hierarchical clustering heatmap analysis of these phenolic compounds showed that metabolite levels were significantly upregulated under AQ3 treatment. For the aboveground parts, AQ3 significantly increased the levels of metabolites such as Hesperetin, Astragalin, Luteolin, and Chrysin. For the below-ground parts, AQ3 significantly increased the levels of metabolites including Naringenin, Kaempferol, and Isoliquiritigenin. Additionally, AQ3 significantly elevated the levels of L-Phenylalanine, Apigenin, Cinnamic acid, and Galangin in both the aboveground and belowground parts of the plant (Figure 4C,F).

### 3.6. Transcriptome Sequencing and Enrichment Analysis of DEGs

Based on the above analyses, significant differences were observed in physiological growth, primary metabolism, and flavonoid-related traits of *A. membranaceus* under AQ3 treatment. To further elucidate the molecular mechanisms underlying these effects, transcriptome sequencing was performed on leaves and roots of *A. membranaceus* treated with AQ3 and compared with the control (CK).

**Figure 5:**
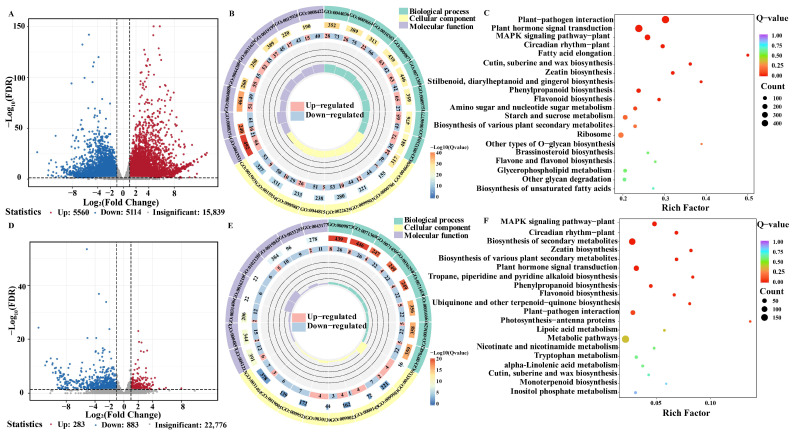
Differential gene screening and GO and KEGG enriched pathways of *A. membranaceus* after different microbial treatments. (**A**) Volcano plot of DEGs in *A. membranaceus* leaves; (**B**) GO enrichment analysis of DEGs in *A. membranaceus* leaves; (**C**) KEGG pathway enrichment analysis of DEGs in *A. membranaceus* leaves; (**D**) Volcano plot of DEGs in *A. membranaceus* roots; (**E**) GO enrichment analysis of DEGs in *A. membranaceus* roots; (**F**) KEGG pathway enrichment analysis of DEGs in *A. membranaceus* roots.

Differentially expressed genes (DEGs) were identified based on read count data using the criteria of |log_2_ fold change| ≥ 2 and false discovery rate (FDR) < 0.05 in both leaves and roots under AQ3 treatment and CK conditions. Compared with the control, a total of 10,674 DEGs were identified in leaves, including 5560 upregulated and 5114 downregulated genes. In roots, a total of 1166 DEGs were detected, comprising 283 upregulated and 883 downregulated genes (Figure 5A,D).

GO annotations were classified into three categories: molecular function, biological process, and cellular component. The top eight GO terms with the lowest q-values in each category were selected for visualization. In leaves, the most significantly enriched molecular function term was anchored component of plasma membrane (GO: 0046658), containing 94 DEGs. For biological processes, the cell wall macromolecule metabolic process (GO: 0044036) was significantly enriched, encompassing 101 DEGs. Within the cellular component category, ADP binding (GO: 0043531) showed significant enrichment, including 115 DEGs, of which 94 were upregulated and 21 were downregulated.

In roots, significant enrichment was observed for dioxygenase activity (GO: 0051213), ethylene-activated signaling pathway (GO: 0009873), and phagocytic vesicle (GO: 0045335), which contained 18, 34, and 4 DEGs, respectively (Figure 5B,E).

To further characterize the biological functions of DEGs between AQ3-treated and control plants, KEGG pathway enrichment analysis was conducted. Compared with the control, AQ3 treatment significantly affected multiple signaling regulation and metabolic pathways. In leaves, the plant–pathogen interaction pathway (ko04626) was significantly enriched, comprising 475 genes, including 360 upregulated and 115 downregulated genes. In the category of environmental information processing, plant hormone signal transduction (ko04075) and MAPK signaling pathway–plant (ko04016) were significantly enriched. The plant hormone signal transduction pathway included 466 genes, with 227 upregulated and 239 downregulated genes, while the MAPK signaling pathway–plant consisted of 216 genes (140 upregulated and 76 downregulated).

Among metabolism-related pathways, phenylpropanoid biosynthesis (ko00940) and flavonoid biosynthesis (ko00941) were significantly enriched. In the phenylpropanoid biosynthesis pathway, 51 genes were upregulated and 45 genes were downregulated, while the flavonoid biosynthesis pathway contained 39 genes, including 21 upregulated and 18 downregulated genes. These results further indicate that the microbial consortium AQ3 can regulate the growth and development of *A. membranaceus* through modulation of secondary metabolism (Figure 5C).

In roots, circadian rhythm–plant (ko04712), biosynthesis of secondary metabolites (ko01110), and zeatin biosynthesis (ko00908) pathways were also significantly enriched (Figure 5F).

### 3.7. Integrated Transcriptomic and Metabolomic Analyses Reveal the Regulatory Mechanisms of Microbial Treatment on Flavonoid Biosynthesis in A. membranaceus

Based on the integrated analysis of KEGG pathway enrichment and flavonoid biosynthetic pathways, this study systematically elucidated the regulatory mechanisms by which the microbial consortium modulates flavonoid biosynthesis in *A. membranaceus*. The results showed that AQ3 treatment significantly increased the content of L-phenylalanine in the phenylalanine metabolism pathway.

At the molecular level, this process was primarily regulated by phenylalanine ammonia-lyase (*PAL*) genes. Six key differentially expressed *PAL* genes (*IMPGAME1N346025*, *IMPGAME1N346237*, *IMPGAME1N346217*, *IMPGAME1N346233*, *IMPGAME1N346042*, and *IMPGAME1N346061*) were significantly upregulated under AQ3 treatment. The upregulation of *PAL* genes may have contributed to the accumulation of cinnamic acid, while the downstream Cinnamate 4-Hydroxylase (C4H) and 4-coumarate-CoA ligase (*4CL*) genes (*IMPGAME1N125282* and *IMPGAME1N314990*) also showed increased expression under AQ3 treatment.

**Figure 6:**
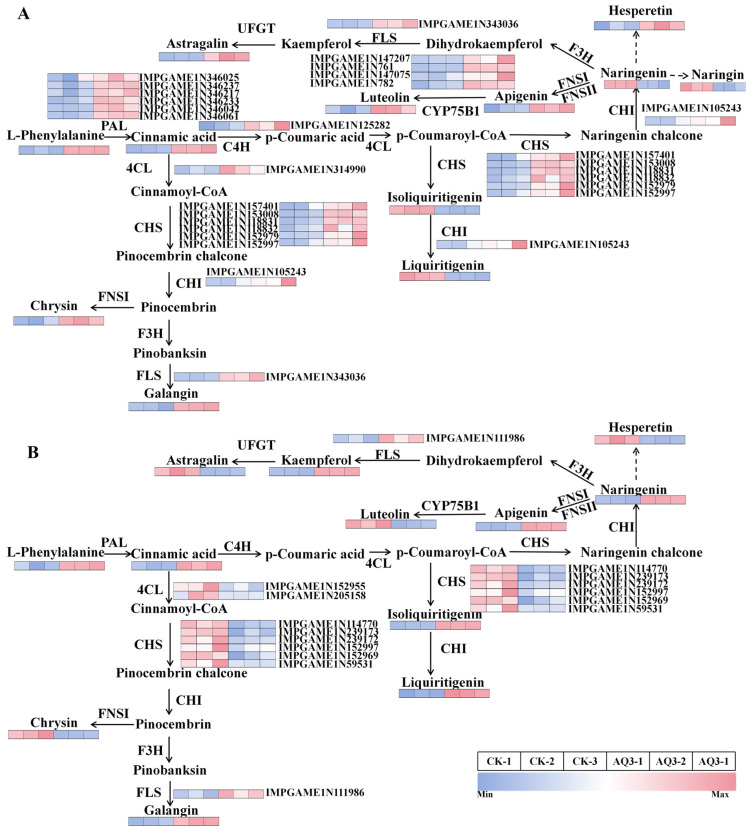
Changes in related metabolites and differential genes in flavonoid biosynthesis pathway of leaves (**A**) and roots (**B**) of *A. membranaceus* treated with different microbial agents. Dashed arrows represent multi-step processes.

Further analysis of metabolite contents and differentially expressed genes involved in the flavonoid biosynthesis pathway between AQ3-treated and control (CK) plants is shown in Figure 6. In leaves, the upregulation of the flavonol synthase (*FLS*) gene (*IMPGAME1N343036*) markedly promoted the accumulation of galangin under AQ3 treatment (Figure 6A). In roots, the corresponding *FLS* gene (*IMPGAME1N111986*) was also significantly upregulated, contributing to increased galangin accumulation under AQ3 treatment (Figure 6B).

Under AQ3 treatment, the accumulation of luteolin in leaves was enhanced through the conversion of apigenin, which was mediated by the cytochrome P450 enzyme (*CYP75B1*) encoded by four upregulated genes (*IMPGAME1N147207*, *IMPGAME1N761*, *IMPGAME1N147075*, and *IMPGAME1N782*) (Supplementary File S5).

## 4. Discussion

### 4.1. Colonization of A. membranaceus Roots as Affected by Different Microbial Agents

Root colonization is a prerequisite for microorganisms to exert their growth-promoting functions [35]. The results of this study showed that both single and dual inoculations of AMF and *C. globosum* successfully colonized the roots of *A. membranaceus*, but the colonization rates varied significantly between treatments, exhibiting clear dose-dependent and interaction effects. Under single inoculation with *C. globosum*, the root colonization rate increased with higher inoculation concentrations, reaching its maximum in the Q3 treatment (10^8^ spores/mL). This suggests that, within a certain range, increasing the inoculation dose of *C. globosum* promotes its successful colonization in the rhizosphere and roots of *A. membranaceus*, consistent with the findings of Xie et al. [36].

Notably, in dual inoculation treatments, the presence of AMF significantly altered the colonization behavior of *C. globosum*. In AQ1 and AQ2 treatments, the colonization rate of *C. globosum* was higher than in the single inoculation treatments at the same concentrations, indicating that at low to medium concentrations, AMF may improve the rhizosphere microenvironment, increase root exudates, or expand the hyphal network, providing more favorable colonization conditions for *C. globosum*. Previous studies have shown that AMF can alter the composition of root exudates and modify the bacterial community structure in the rhizosphere [37]. However, in the AQ3 treatment, the colonization rate of *C. globosum* was lower than in Q3, suggesting that at high concentrations of *C. globosum*, there may be some degree of spatial or resource competition between AMF and *C. globosum* [38].

In contrast to *C. globosum*, the colonization rate of AMF in dual inoculation treatments was significantly higher than that in single AMF inoculation, reaching its maximum value in AQ3, with a 66.17% increase compared to single AMF inoculation. This result indicates that the presence of *C. globosum* overall promoted the colonization and expansion of AMF in the roots of *A. membranaceus*.

Studies have shown that under suitable concentration combinations (especially AQ3), a stable symbiotic system is formed, with AMF as the dominant partner and *C. globosum* as an auxiliary, laying the microbial foundation for subsequent promotion of aboveground growth and metabolic reprogramming.

### 4.2. Synergistic Promotion of A. membranaceus by AMF and C. globosum: Growth, Active Compounds, and Photosynthesis

In this study, all microbial treatments promoted the growth and development of *A. membranaceus* to varying degrees, but the growth-promoting effect of the dual inoculation treatments was significantly stronger than that of the single inoculation treatments. Specifically, AQ3 showed overwhelming advantages in plant height, root length, aboveground and underground biomass. These results indicate that the growth promotion of *A. membranaceus* by AMF and *C. globosum* is not a simple additive effect, but rather a significant synergistic effect generated through functional complementarity. This view is supported by a study on perennial ryegrass, which showed that dual inoculation with AMF and an endophytic fungus significantly improved the growth of perennial ryegrass [39]. A study found that dual inoculation with AMF and plant growth-promoting rhizobacteria (PGPR) significantly improved the rhizosphere microbial community structure and function, promoting the growth of tobacco seedlings [40].

From the perspective of root systems, AMF significantly expands the root’s absorption range for water and mineral nutrients, particularly phosphorus, through its hyphal network [41]. In contrast, *C. globosum* may enhance root activity and absorption efficiency by secreting plant hormone analogs or promoting root branching. For example, the endophytic fungus *C. globosum* strain ND35 promotes cucumber growth through complex plant hormone biosynthesis and metabolic regulation [14]. *C. globosum* strain D38 forms a mutualistic interaction with *Salvia miltiorrhiza*, promoting overall plant growth and secondary metabolism, thus enhancing the accumulation of salvianolic acids and tanshinonesc [42]. Therefore, the combined action of AMF and *C. globosum* enables *A. membranaceus* to form a more developed and efficient root system, providing continuous material and energy support for aboveground growth.

In this study, the greenhouse environment was not strictly controlled, with temperature and humidity fluctuating between 20–26 °C and 65–90%, respectively. It should be noted that the temperature range (20–26 °C) employed in this study is generally favorable for the growth of *A*. *membranaceus* and AMF colonization [43]. Previous studies have reported that the optimal temperature for mycorrhizal symbiosis in medicinal plants is typically 20–25 °C, and the temperature conditions in this experiment largely fell within this suitable range [44]. However, the relatively high humidity (65–90%) may have altered nutrient uptake efficiency by affecting transpiration. Under high humidity conditions, the water vapor pressure deficit between the leaf and the atmosphere decreases, which reduces transpiration-driven mass flow, potentially decreasing the migration rate of soluble nutrients (e.g., N, P, K) from the soil to the root surface. This may partially explain the unstable growth performance observed in the non-inoculated treatment groups under high temperature and humidity conditions. Notably, despite the environmental fluctuations, the AQ3 treatment consistently exhibited significant and stable growth-promoting effects throughout the experimental period. This result suggests that the AQ3 microbial consortium may enhance host plant stress tolerance, helping plants buffer against the stress caused by environmental fluctuations. Previous studies have confirmed that certain beneficial microorganisms can improve plant tolerance to temperature and humidity fluctuations by modulating plant antioxidant systems, osmoregulatory substances, and hormone signaling pathways [45].

At the secondary metabolism level, numerous studies have reported the important role of microorganisms in regulating the synthesis of plant secondary metabolites [46,47]. For example, the endophytic fungus *Pseudodidymocyrtis lobariellae* KL27 promotes the growth and development of *Taxus* and influences the biosynthesis and accumulation of paclitaxel [48].

The combined microbial inoculants had a particularly significant effect on the promotion of bioactive compounds in *A. membranaceus*. This study found that the content of calycosin-7-glucoside and cycloastragenol in the aboveground part of *A. membranaceus* significantly increased in AQ2 and AQ3 treatments. In the underground part, calycosin-7-glucoside and several astragalosides reached their maximum levels in AQ3 treatment. The increased content of calycosin-7-glucoside helps enhance its secondary metabolite reserve capacity, improving the safe storage and regulatory efficiency of bioactive compounds in the plant, participating in stress response and defense-related metabolic processes, and enhancing the quality and functional component base of *A. membranaceus* for medicinal purposes [49,50].

Changes in photosynthetic parameters further support this view. Photosynthesis is one of the key physiological and metabolic activities in plants, closely related to upstream and downstream physiological processes [51]. Dual inoculation treatments, especially AQ3, significantly increased the net photosynthetic rate (Pn), stomatal conductance (Gs), and transpiration rate (Tr) of *A. membranaceus*, while also enhancing PSII actual photochemical efficiency (Y (II)) and electron transport rate (ETR). This indicates that the combined microbial inoculants not only improved photosynthetic carbon assimilation but also optimized light energy utilization efficiency, making the light and dark reactions more coordinated [52].

Additionally, the increase in NPQ and Fv/Fo in AQ3 treatment suggests that the combined microbial inoculants enhanced the regulation and utilization of light energy in *A. membranaceus*, reducing the risk of photoinhibition. This overall enhancement in photosynthetic performance is in line with the significant increase in biomass observed in subsequent measurements.

### 4.3. Combined Microbial Inoculants Regulate Antioxidant System in A. membranaceus

The antioxidant system is an important defense mechanism for plants to cope with environmental changes and maintain cellular homeostasis. It includes both enzymatic and non-enzymatic components that protect plants from stress by scavenging excess reactive oxygen species (ROS) [53,54]. This study found that after applying AMF and *C. globosum*, the activity of several antioxidant enzymes (SOD, POD, CAT, APX) in *A. membranaceus* was significantly increased, with the enhancement generally greater in dual inoculation treatments, especially in AQ3.

The significant increase in SOD and POD activities helps to rapidly scavenge superoxide anions and peroxides, preventing the accumulation of ROS within the cell. The synergistic action of CAT and APX further reduces H_2_O_2_ levels [28]. Correspondingly, in the AQ3 treatment, MDA content and superoxide anion production rate were significantly reduced, indicating that the combined microbial inoculants effectively alleviated lipid peroxidation damage, maintaining the integrity of the cell membrane, which is consistent with previous studies [55,56].

Notably, there are differences in the response to microbial inoculants between different organs. In the underground part, the increase in SOD and POD activities in AQ3 treatment was particularly significant, indicating that the combined microbial inoculants primarily enhanced the antioxidant defense capacity of the roots.

### 4.4. Transcriptomic and Metabolomic Insights into Microbial Consortium-Mediated Accumulation of Medicinal Compounds

Flavonoids are among the most important bioactive medicinal constituents in *A. membranaceus*. Their biosynthesis initiates from the phenylalanine metabolic pathway and is finely regulated by key rate-limiting enzymes and complex transcriptional regulatory networks. By integrating transcriptomic and metabolomic datasets, the present study systematically elucidated the regulatory mechanisms by which the microbial consortium, particularly the AQ3 treatment, modulates flavonoid biosynthesis in *A. membranaceus*. Our results demonstrate that this regulatory process does not rely on the enhancement of a single metabolic node but instead involves multilayered and coordinated regulation spanning from upstream precursor supply to downstream structural modification steps [57].

At the upstream level of the metabolic pathway, AQ3 treatment significantly increased the content of L-phenylalanine and was accompanied by the pronounced upregulation of multiple *PAL* genes, indicating that the microbial consortium initially enhances substrate availability for flavonoid biosynthesis at the metabolic source. As a key rate-limiting enzyme that channels phenylalanine metabolism toward secondary metabolism, *PAL* is widely regarded as an important indicator of a plant’s potential for flavonoid and related phenylpropanoid biosynthesis. The coordinated upregulation of multiple *PAL* genes suggests that AQ3 treatment does not induce an isolated gene response but rather activates the *PAL* gene family at a systemic level. This collective transcriptional activation strengthens metabolic flux toward secondary metabolism, thereby establishing a robust metabolic foundation for subsequent flavonoid scaffold biosynthesis.

Moreover, the upregulation trend observed for downstream *4CL* genes further supports the notion that the phenylpropanoid metabolic pathway is globally activated under AQ3 treatment.

In the middle and downstream sections of the flavonoid biosynthesis pathway, organ-specific regulatory differences were observed; however, an overall regulatory pattern characterized by the “upregulation of key structural enzyme expression and the directed accumulation of specific flavonoid monomers” was evident. In both leaves and roots, the significant upregulation of *FLS* genes was synchronously accompanied by increased galangin accumulation, indicating that the microbial consortium enhances the activity of the flavonol branch pathway and thereby promotes the accumulation of flavonols with high biological activity [58].

As a key enzyme controlling the conversion of dihydroflavonols to flavonols, enhanced *FLS* expression generally reflects a metabolic shift in secondary metabolism toward flavonol compounds with stronger antioxidant capacity. This observation is highly consistent with the results of the present study, in which the microbial consortium significantly strengthened the antioxidant defense system of *A. membranaceus*.

Moreover, under AQ3 treatment, the significant upregulation of the *CYP75B1* gene family in leaves drove the hydroxylation of apigenin to luteolin, highlighting the directed regulatory role of the microbial consortium at the “fine structural modification stage” of flavonoid biosynthesis. *CYP75B1* encodes flavonoid 3′-hydroxylase, a key enzyme determining the degree of hydroxylation and biological activity of flavonoids. Its upregulation not only increased the accumulation of highly bioactive flavonoids such as luteolin but may also enhance their functional contributions to antioxidative defense, stress resistance, and the formation of medicinal quality.

Collectively, the microbial consortium simultaneously enhanced phenylalanine supply, activated the expression of key rate-limiting enzyme genes, and directed flavonoid biosynthesis toward specific high-activity branches, thereby establishing a coordinated regulatory mode characterized by “precursor reinforcement, flux amplification, and structural optimization” [59]. The high concordance between transcriptional regulation and metabolic responses clearly demonstrates that the combined application of AMF and *C. globosum* does not merely passively promote metabolite accumulation, but rather actively remodels the secondary metabolic network of *A. membranaceus*, enabling the efficient biosynthesis and targeted accumulation of flavonoid bioactive compounds [60]. This finding provides clear molecular mechanistic evidence for microbial inoculant-mediated regulation of secondary metabolism in medicinal plants and offers a novel theoretical basis for the precise improvement of medicinal quality in *A. membranaceus* [61].

## 5. Conclusions

The microbial consortium AQ3 significantly promoted root colonization by AMF and optimized the synergistic interaction between AMF and *C. globosum* in the rhizosphere, with no observable colonization antagonism. These two beneficial microorganisms may cooperatively enhance root function through resource complementarity and signaling coordination, thereby improving nutrient acquisition efficiency and environmental adaptability of *A. membranaceus*.

AQ3 treatment significantly elevated the activities of antioxidant enzymes (SOD and POD), with a stronger response magnitude in roots than in aboveground tissues, indicating that the microbial consortium preferentially reinforced the root antioxidant defense system, contributing to the mitigation of environmental stress and the maintenance of cellular homeostasis.

Integrated multi-omics analysis revealed that AQ3 treatment markedly upregulated key structural genes involved in the phenylalanine metabolism and flavonoid biosynthesis pathways, including *PAL*, *4CL*, *FLS*, and *CYP75B1*. This upregulation promoted the metabolic flux from L-phenylalanine toward downstream flavonoid metabolites, driving the accumulation of major flavonoids such as galangin and luteolin in both roots and leaves. The high consistency between transcriptional changes and metabolite accumulation reveals a molecular regulatory mechanism of “microbe–gene expression–metabolic reprogramming” through which the microbial consortium modulates the formation of medicinal compounds.

In conclusion, the microbial consortium AQ3 synergistically enhanced the growth and medicinal quality of *A. membranaceus* by reinforcing rhizosphere symbiosis, improving antioxidant defense capacity, and reshaping the flavonoid metabolic network.

## Data Availability

The nucleotide sequence generated in this study has been deposited in NCBI GenBank under accession number PZ273390. The sequencing data have been deposited in the NCBI Sequence Read Archive (SRA) under the BioProject accession number PRJNA1434647 [National Center for Biotechnology Information: https://www.ncbi.nlm.nih.gov/, accessed on 31 March 2026] repository.

## Supplementary Materials

The following supporting information can be downloaded at: https://www.mdpi.com/article/10.3390/metabo16050313/s1. Supplementary File S1: Daily fluctuations of temperature and humidity during the experimental period; Supplementary File S2: The complete ANOVA results for the relevant indicators; Supplementary File S3: Table S1: Photosynthetic Parameters of *A*. *membranaceus* Leaves under Different Treatment; Table S2: Chlorophyll Fluorescence Parameters of *A. membranaceus* Leaves under Different Treatment; Supplementary File S4: Basic information of 39 compounds determined by LC-MS; Supplementary File S5: Expression changes of genes in the transcriptome.

## Abbreviations

The following abbreviations are used in this manuscript:

AMF	Arbuscular mycorrhizal fungi
ANOVA	Analysis of variance
APX	Ascorbate peroxidase
CAT	Catalase
cDNA	Complementary DNA
CFU	Colony-forming unit
Ci	Intercellular CO_2_ concentration
*CYP75B1*	Cytochrome P450 75B1 (flavonoid 3′-hydroxylase)
*C4H*	Cinnamate 4-hydroxylase
DEG	Differentially expressed gene
DEM	Differentially expressed metabolite
ETR	Electron transport rate
FDR	False discovery rate
*FLS*	Flavonol synthase
FPKM	Fragments Per Kilobase of transcript per Million mapped reads
Fv/Fo	Potential photochemical activity of PSII
GO	Gene Ontology
Gs	Stomatal conductance
KEGG	Kyoto Encyclopedia of Genes and Genomes
LC–MS	Liquid chromatography–mass spectrometry
MDA	Malondialdehyde
NPQ	Non-photochemical quenching
O_2_^.−^	Superoxide anion
*PAL*	Phenylalanine ammonia-lyase
PCA	Principal component analysis
PDA	Potato dextrose agar
POD	Peroxidase
Pn	Net photosynthetic rate
PSII	Photosystem II
qP	Photochemical quenching coefficient
RNA	Ribonucleic acid
ROS	Reactive oxygen species
SOD	Superoxide dismutase
Tr	Transpiration rate
Y(II)	Actual photochemical efficiency of PSII
*4CL*	4-Coumarate-CoA ligase
