# Supplementary material for: Physiology, Metabolomics, and Transcriptomics Reveal Effects of AMF and Chaetomium globosum Co-Inoculation on Growth and Medicinal Compounds in Astragalus membranaceus
Source: Metabolites. 2026 May 3;16(5):313. doi: 10.3390/metabo16050313 (PMC13208801; doi:10.3390/metabo16050313)
Supplement: Supplementary file 1 [file metabolites-16-00313-s001.zip › Supplementary File S1.pdf]

Daily fluctuations of temperature and humidity during the experimental period

Day	Temperature （℃）	Relative Humidity (%)
1	20.3	88
2	20.5	78
3	20.8	86
4	21	85
5	21.2	84
6	21.4	73
7	21.3	84
8	21.6	82
9	21.9	81
10	22.1	80
11	22.3	79
12	22.5	87
13	22.4	85
14	22.7	77
15	22.9	76
16	23.1	75
17	23	76
18	23.3	74
19	23.5	83
20	23.7	72
21	23.9	71
22	24	70
23	24.2	69
24	24.1	70
25	23.8	72
26	23.6	73
27	23.4	74
28	23.2	75
29	23	76
30	23.3	74
31	23.5	75
32	23.8	73
33	24	72
34	24.2	71
35	24.5	70
36	24.3	71
37	24.1	72
38	24.4	70
39	24.6	69
40	24.8	68
41	25	67
42	24.9	68

43	25.1	67
44	25.3	66
45	25.2	67
46	25.4	66
47	25.5	65
48	25.3	66
49	25.1	67
50	25	68
51	24.8	69
52	24.6	70
53	24.5	71
54	24.3	72
55	24.1	73
56	23.9	74
57	23.8	75
58	24	73
59	24.3	71
60	24.5	70
61	24.2	71
62	24	72
63	23.8	74
64	23.9	73
65	23.7	74
66	23.5	75
67	23.6	74
68	23.8	73
69	24	72
70	24.1	71
71	24.3	70
72	24.5	69
73	24.6	68
74	24.4	69
75	24.2	70
76	24	71
77	23.9	72
78	23.7	73
79	23.5	74
80	23.6	73
81	23.8	72
82	24	71
83	24.2	70
84	24.4	69
85	24.5	68
86	24.3	69

87	24.1	70
88	23.9	71
89	23.7	72
90	23.5	73
