# Supplementary material for: Physiology, Metabolomics, and Transcriptomics Reveal Effects of AMF and Chaetomium globosum Co-Inoculation on Growth and Medicinal Compounds in Astragalus membranaceus
Source: Metabolites. 2026 May 3;16(5):313. doi: 10.3390/metabo16050313 (PMC13208801; doi:10.3390/metabo16050313)
Supplement: Supplementary file 1 [file metabolites-16-00313-s001.zip › Supplementary File S2.pdf]

Treatment (plant height)	Shapiro-Wilk test p value	Brown-Forsythe test p value	ANOVA	Cv(%)
CK	0.0712	0.4166	F (7, 40) = 109.1 P<0.0001	6.63%
Q1	0.2085			12.71%
Q2	0.8643			4.79%
Q3	0.9047			7.80%
AMF	0.5473			4.29%
AQ1	0.5164			5.08%
AQ2	0.7277			5.46%
AQ3	0.6654			7.08%

Treatment (AMF colonization rate)	Shapiro-Wilk test p value	Brown-Forsythe test p value	ANOVA	Cv(%)
AMF	0.9559	0.7332	F (3, 8) = 21.10 P=0.0004	9.99%
AQ1	0.5026			4.30%
AQ2	0.7162			7.66%
AQ3	0.9056			8.42%

Treatment (*C. globosum* colonization rate)	Shapiro-Wilk test p value	Brown-Forsythe test p value	ANOVA	Cv(%)
Q1	0.4144	0.7613	F (5, 12) = 23.47 P<0.0001	14.96%
Q2	0.6642			8.76%
Q3	0.6878			9.98%
AQ1	0.5026			10.04%
AQ2	0.7494			18.85%
AQ3	0.6178			9.10%

Treatment (POD activity in aboveground parts)	Shapiro-Wilk test p value	Brown-Forsythe test p value	ANOVA	Cv(%)
CK	0.2602	0.9260	F (7, 16) = 232.6 P<0.0001	4.01%
Q1	0.5928			2.70%
Q2	0.9420			2.69%
Q3	0.6788			2.63%
AMF	0.2855			3.30%
AQ1	0.1939			1.32%
AQ2	0.1736			0.87%
AQ3	0.0580			0.86%

Treatment (POD activity in underground parts)	Shapiro-Wilk test p value	Brown-Forsythe test p value	ANOVA	Cv(%)
CK	>0.9999	0.8809	F (7, 16) = 501.6 P<0.0001	10.84%
Q1	0.5542			4.30%
Q2	0.5163			5.93%
Q3	0.4335			2.07%
AMF	0.6369			2.03%
AQ1	0.7120			3.97%
AQ2	0.2103			2.52%
AQ3	0.2479			1.24%

Treatment (MDA contents in aboveground parts)	Shapiro-Wilk test p value	Brown-Forsythe test p value	ANOVA	Cv(%)
CK	0.4619	0.5884	F (7, 16) = 79.96 P<0.0001	1.95%
Q1	0.6091			4.81%
Q2	0.6930			9.75%
Q3	0.6397			5.67%
AMF	0.3562			11.73%
AQ1	0.8710			1.81%
AQ2	0.2154			4.53%
AQ3	0.7490			7.95%

Treatment (MDA contents in underground parts)	Shapiro-Wilk test p value	Brown-Forsythe test p value	ANOVA	Cv(%)
CK	0.7482	0.1618	F (7, 16) = 74.55 P<0.0001	7.67%
Q1	0.9107			12.11%
Q2	0.9767			1.10%
Q3	0.8275			4.22%
AMF	0.7007			2.35%
AQ1	0.4204			4.53%
AQ2	0.5312			4.32%
AQ3	0.4639			8.75%

Treatment (APX activity in aboveground parts)	Shapiro-Wilk test p value	Brown-Forsythe test p value	ANOVA	Cv(%)
CK	0.9475	0.3200	F (7, 16) = 15.84 P<0.0001	40.92%
Q1	0.6970			5.27%
Q2	0.9265			8.19%
Q3	0.7729			23.00%
AMF	0.4633			4.03%
AQ1	>0.9999			5.26%
AQ2	0.5015			16.08%
AQ3	0.8934			9.41%

Treatment (APX activity in underground parts)	Shapiro-Wilk test p value	Brown-Forsythe test p value	ANOVA	Cv(%)
CK	0.9213	0.6463	F (7, 16) = 28.45 P<0.0001	4.88%
Q1	0.4537			16.38%
Q2	0.3472			4.04%
Q3	0.1965			8.95%
AMF	0.9069			13.07%
AQ1	>0.9999			4.17%
AQ2	0.9265			8.90%
AQ3	>0.9999			16.22%

Index	Kruskal-Wallis test p value
Root length	<0.0001
Fresh weight of aboveground parts	<0.0001
Fresh weight of underground parts	<0.0001
Dry weight of aboveground parts	<0.0001
Dry weight of underground parts	<0.0001
SOD activity in aboveground parts	0.0021
SOD activity in underground parts	0.0020
CAT activity in aboveground parts	0.0020
CAT activity in underground parts	0.0026
$O2^{\cdot-}$ production rate in aboveground parts	0.0020

$O2^{\cdot-}$ production rate in underground parts	0.0019
