# Supplementary material for: Physiology, Metabolomics, and Transcriptomics Reveal Effects of AMF and Chaetomium globosum Co-Inoculation on Growth and Medicinal Compounds in Astragalus membranaceus
Source: Metabolites. 2026 May 3;16(5):313. doi: 10.3390/metabo16050313 (PMC13208801; doi:10.3390/metabo16050313)
Supplement: Supplementary file 1 [file metabolites-16-00313-s001.zip › Supplementary File S3.pdf]

Table S1. Photosynthetic Parameters of *A. membranaceus* Leaves under Different Treatment.

Treatment	Pn（μmol· m $^{-2}$· s $^{-1}$）	Pn Cv (%)	Gs（μmol· m $^{-2}$· s $^{-1}$）	Gs Cv (%)	Ci（μmol· m $^{-2}$· s $^{-1}$）	Ci Cv (%)	Tr (μmol· m $^{-2}$· s $^{-1}$)	Tr Cv (%)
CK	4.653±0.145 f	3.12%	0.041±0.001 d	1.97%	213.667±2.082 e	0.97%	0.880±0.026 e	3.01%
Q1	5.290±0.056 e	1.05%	0.047±0.000 c	0.44%	204.333±3.512 e	1.72%	0.982±0.002 d	0.21%
Q2	5.615±0.145 e	2.58%	0.048±0.000 c	0.44%	235.5±1.323 cd	0.56%	0.987±0.003 d	0.29%
Q3	6.530±0.062 d	0.96%	0.063±0.006 b	8.99%	229.334±3.215 d	1.40%	1.543±0.021 b	1.35%
AMF	7.233±0.232 c	3.20%	0.094±0.000 a	0.52%	241.667±0.577 c	0.24%	1.447±0.035 c	2.43%
AQ1	8.300±0.332 b	3.99%	0.095±0.000 a	0.34%	244.333±2.082 c	0.85%	1.877±0.006 a	0.31%
AQ2	8.600±0.031 ab	0.36%	0.095±0.000a	0.11%	283.333±7.572 a	2.67%	1.880±0.010 a	0.53%
AQ3	9.103±0.228 a	2.51%	0.095±0.000 a	0.07%	261.000±7.550 b	2.89%	1.890±0.000 a	0.00%
ANOVA		$F (7, 40) = 496.4$ $P<0.0001$		$F (7, 40) = 889.3$ $P<0.0001$		$F (7, 40) = 205.8$ $P<0.0001$		$F (7, 40) = 3619$ $P<0.0001$

Table S2 Chlorophyll Fluorescence Parameters of *A. membranaceus* Leaves under Different Treatment

Treatment	Fv/Fo	Fv/Fo Cv (%)	Fv/Fm	Fv/Fm Cv (%)	Y (II)	Y (II) Cv (%)	qP	qP Cv (%)	NPQ	NPQ Cv (%)	ETR	ETR Cv (%)
CK	3.449±0.134 b	3.90%	0.764±0.013 b	1.74%	0.592±0.005e	0.93%	0.836±0.013 b	1.55%	0.172±0.029 c	17.06%	24.9±0.36 e	1.45%
Q1	3.783±0.293 ab	7.74%	0.804±0.017 a	2.06%	0.633±0.023 d	3.57%	0.876±0.026 ab	2.95%	0.210±0.005 bc	2.19%	26.567±0.971 d	3.65%
Q2	4.112±0.306 ab	7.43%	0.804±0.012 a	1.44%	0.659±0.004 c	0.68%	0.935±0.051 ab	5.48%	0.246±0.01 bc	0.41%	27.7±0.2 c	0.72%
Q3	4.303±0.339 a	7.87%	0.811±0.012 a	1.48%	0.673±0.004 bc	0.56%	0.851±0.088 ab	10.34%	0.223±0.003 bc	1.38%	28.234±0.153 bc	0.54%
AMF	4.073±0.274 ab	6.74%	0.803±0.010 a	1.30%	0.681±0.003 ac	0.04%	0.944±0.027 ab	2.81%	0.273±0.006 bc	2.20%	28.634±0.153 ac	0.53%
AQ1	4.208±0.269 a	6.40%	0.808±0.010 a	1.27%	0.694±0.003 ab	0.36%	0.927±0.009 ab	1.02%	0.300±0.014 bc	4.60%	29.1±0.1 ab	0.34%
AQ2	4.353±0.186 a	4.28%	0.790±0.012 ab	1.62%	0.699±0.00 a	0.65%	0.945±0.009 ab	0.90%	0.349±0.023 b	6.63%	29.334±0.153 a	0.52%
AQ3	4.305±0.236 a	5.48%	0.811±0.008 a	1.04%	0.705±0.003 a	0.38%	0.950±0.009 a	0.91%	0.529±0.134 a	25.34%	29.6±0.1 a	0.34%
ANOVA		F (7, 40) = 8.475 P<0.0001		F (7, 40) = 9.907 P<0.0001		F (7, 40) = 115.7 P<0.0001		F (7, 40) = 8.433 P<0.0001		F (7, 40) = 30.66 P<0.0001		F (7, 40) = 101.2 P<0.0001
