# Supplementary material for: Physiology, Metabolomics, and Transcriptomics Reveal Effects of AMF and Chaetomium globosum Co-Inoculation on Growth and Medicinal Compounds in Astragalus membranaceus
Source: Metabolites. 2026 May 3;16(5):313. doi: 10.3390/metabo16050313 (PMC13208801; doi:10.3390/metabo16050313)
Supplement: Supplementary file 1 [file metabolites-16-00313-s001.zip › Supplementary File S4..pdf]

Basic information of 39 compounds determined by LC-MS

Compound	Chemical formula
Abscisic Acid	$C_{15}H_{20}O_4$
Apigenin	$C_{15}H_{10}O_5$
Astragalin	$C_{21}H_{20}O_{11}$
Astragaloside I	$C_{45}H_{72}O_{16}$
Astragaloside II	$C_{43}H_{70}O_{15}$
Astragaloside III	$C_{41}H_{68}O_{14}$
Aucubin	$C_{15}H_{22}O_9$
Calycosin-7-glucoside	$C_{22}H_{22}O_{10}$
Catechin	$C_{15}H_{14}O_6$
Chlorogenic acid	$C_{16}H_{18}O_9$
Chrysin	$C_{15}H_{10}O_4$
Cinnamic acid	$C_9H_8O_2$
Cycloastragenol	$C_{30}H_{50}O_5$
Ferulic acid	$C_{10}H_{10}O_4$
Galangin	$C_{15}H_{10}O_5$
Genistein	$C_{15}H_{10}O_5$
Genistin	$C_{21}H_{20}O_{10}$
Gentisic acid	$C_7H_6O_4$
Hesperetin	$C_{16}H_{14}O_6$
Hyperoside	$C_{21}H_{20}O_{12}$
Isofraxidin	$C_{11}H_{10}O_5$
Isoliquiritigenin	$C_{15}H_{12}O_4$
Isoquercitrin	$C_{21}H_{20}O_{12}$
Kaempferol	$C_{15}H_{10}O_6$
Liquiritigenin	$C_{15}H_{12}O_4$
Loganin	$C_{17}H_{26}O_{10}$
Luteolin	$C_{15}H_{10}O_6$
L-Phenylalanine	$C_9H_{11}NO_2$
Naringenin	$C_{15}H_{12}O_5$
Naringin	$C_{27}H_{32}O_{14}$
Oleanolic Acid	$C_{30}H_{48}O_3$
Quercitrin	$C_{21}H_{20}O_{11}$
Rutin	$C_{27}H_{30}O_{16}$
Sinapic Acid	$C_{11}H_{12}O_5$
Syringic acid	$C_9H_{10}O_5$
Syringin	$C_{17}H_{24}O_9$
Tabersonine	$C_{21}H_{24}N_2O_2$
Trigonelline	$C_7H_7NO_2$
Tryptamine	$C_{10}H_{12}N_2$
